# Ser or Leu: structural snapshots of mistranslation in *Candida albicans*

**DOI:** 10.3389/fmolb.2014.00027

**Published:** 2014-12-19

**Authors:** Zsuzsa Sárkány, Alexandra Silva, Pedro J. B. Pereira, Sandra Macedo-Ribeiro

**Affiliations:** ^1^Protein Crystallography Group, IBMC – Instituto de Biologia Molecular e Celular, Universidade do PortoPorto, Portugal; ^2^Biomolecular Structure Group, IBMC – Instituto de Biologia Molecular e Celular, Universidade do PortoPorto, Portugal

**Keywords:** CTG-clade, genetic code, cell signaling, MAPK pathway, cAMP-dependent pathway, morphogenesis

## Abstract

*Candida albicans* is a polymorphic opportunistic fungal pathogen normally residing as commensal on mucosal surfaces, skin and gastrointestinal and genitourinary tracts. However, in immunocompromised patients *C. albicans* can cause superficial mucosal infections or life-threatening disseminated candidemia. A change in physiological conditions triggers a cascade of molecular events leading to morphogenetic alterations and increased resistance to damage induced by host defenses. The complex biology of this human pathogen is reflected in its morphological plasticity and reinforced by the ability to ambiguously translate the universal leucine CUG codon predominantly as serine, but also as leucine. Mistranslation affects more than half of *C. albicans* proteome and it is widespread across many biological processes. A previous analysis of CTG-codon containing gene products in *C. albicans* suggested that codon ambiguity subtly shapes protein function and might have a pivotal role in signaling cascades associated with morphological changes and pathogenesis. In this review we further explore this hypothesis by highlighting the role of ambiguous decoding in macromolecular recognition of key effector proteins associated with the regulation of signal transduction cascades and the cell cycle, which are critical processes for *C. albicans* morphogenic plasticity under a variety of environmental conditions.

## *Candida albicans* adaptation to the host

*Candida albicans* is an opportunistic fungal pathogen of humans, that lives as commensal on the skin and mucosal surfaces of healthy individuals (Odds et al., 2001). In response to changes in environmental conditions, *C. albicans* can convert into a disease-causing pathogen, resulting in oral, gastrointestinal and vaginal candidiasis or ultimately lead to a systemic candidemia in immunocompromised individuals. In fact, candidiasis is among the leading nosocomial infections worldwide, and mortality rates resulting from widespread systemic infections range between 15 and 35% (Papon et al., 2013). An important feature of *C. albicans* that elicits its adaptation to different environmental niches and evasion from threats generated by the host immune defenses is its polymorphic phenotype. *C. albicans* is able to reversibly switch between distinct morphogenic states: the yeast form and the filamentous pseudohyphal or hyphal forms (Sudbery, 2011). Furthermore, *C. albicans* can produce biofilms, which consist of a mixed population of yeast, pseudohyphae and hyphae (Douglas, 2003; Blankenship and Mitchell, 2006) and represent a high risk factor for infection and disease (Douglas, 2002, 2003). Morphological transitions have been correlated with a rapid remodeling of the cell transcriptional program in order to perfectly adapt to microenvironmental changes (Shapiro et al., 2011; Sudbery, 2011; Gow et al., 2012). Albeit some virulence-associated gene products were found to modulate *C. albicans* pathogenicity independently of morphogenesis (Noble et al., 2010), the mechanism of morphological switching has proven its relevance in many facets of *C. albicans* virulence (adhesion, invasion, and bloodstream propagation) (Gow et al., 2012). The diversity required for survival is generated by sequential activation of highly coordinated signaling pathways, in response to multiple stress factors and/or damage induced by antifungal drugs (Shapiro et al., 2011).

## The CTG-clade genetic code: a unique sense-to-sense codon reassignment

Throughout evolution the standard genetic code suffered several alterations both in prokaryotic and eukaryotic organisms. The discovery of these alterations, over the last 30 years, “thawed” the frozen accident hypothesis for the genetic code (Crick, 1968). Identified natural genetic code alterations include the reassignment of the identity of both sense and nonsense codons, as well as codon unassignment (Knight et al., 2001; Miranda et al., 2006; Pouplana et al., 2014). Genetic code flexibility was further supported by its expansion to incorporate selenocysteine (Sec, 21st amino acid), pyrrolysine (Pyl, 22nd amino acid) (Zhang and Gladyshev, 2007) and non-natural amino acids (Chatterjee et al., 2012). Incorporation of Sec has been described in diverse enzymes of all kingdoms, from bacteria to eukaryotes (Bock and Stadtman, 1988). This mechanism of translational recoding evolved in order to overcome the presence of a UGA stop codon within the active site of proteins and prevent the translation of truncated, nonfunctional enzymes. The incorporation of Sec at UGA position, using a selenocysteine-specific tRNA, tRNA^Sec^, is regulated by the presence of a Sec insertion sequence (SECIS) at the stem-loop of the 3′-untranslated region of selenoprotein-encoding mRNAs (Bock et al., 1991; Arner, 2010) and, in higher eukaryotes, also within the mRNAs coding region (Mix et al., 2007). Sec insertion enhances the enzymes' kinetic efficiency when compared to its Cys-containing counterparts (Bock et al., 1991; Arner, 2010). Pyl incorporation has been described in methyltransferases from *Methanosarcineace* species (Srinivasan et al., 2002), *Desulfitobacterium hafniense* (Herring et al., 2007) and symbiotic deltaproteobacterium of the gutless worm *Olavius algarvensis* (Zhang and Gladyshev, 2007) that harbor a UAG stop codon within the active site and a pyrrolysine insertion sequence (PYLIS) downstream in the mRNA. Pyl is incorporated during translation, similar to standard amino acids, due to the presence of a specific tRNA harboring a CUA anticodon, the *PylT*-derived tRNA, and a *pylS* gene, encoding an aminoacyl-tRNA synthetase that charges the tRNA with Pyl (Srinivasan et al., 2002). Insertion of non-natural amino acids has been described in *Mycoplasma capricolum* (Yamao et al., 1985) and in metazoan mitochondria (Yokobori et al., 2001) where the universal stop codon UGA is translated as tryptophan. In several ciliate species the stop codons UAA and UAG were also reassigned to code for glutamine (Tourancheau et al., 1995), while in other representatives (*Euplotes* genus) glutamine codon usage is normal but the UGA stop codon is translated as cysteine (Meyer et al., 1991; Tourancheau et al., 1995). Furthermore, [*PSI*+], a non-chromosomal gene of *Saccharomyces cerevisiae*, was shown to be a prion of Sup35, a translation-termination factor of *S. cerevisiae* (Tuite and Cox, 2007). Similar to *sup35* mutations, the [PSI+] prion results in increased read-through of termination codons.

**Figure 1:**
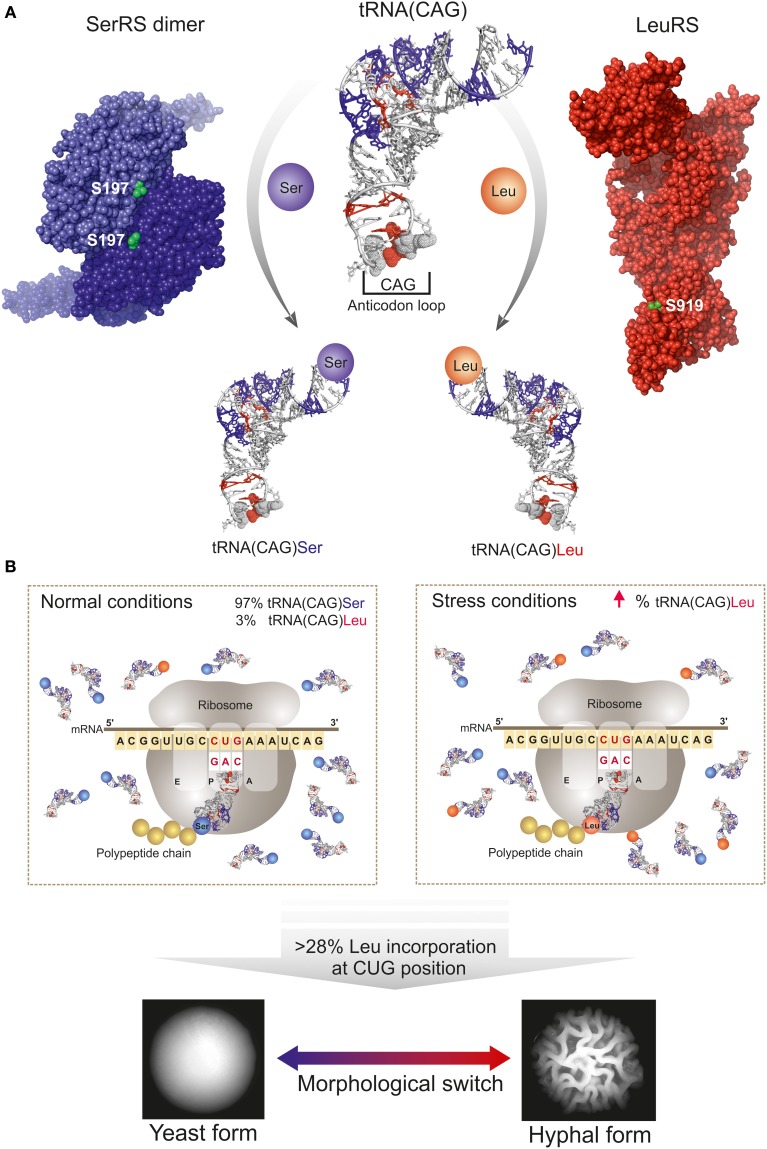
**Schematic representation of CUG codon translation ambiguity mechanism in *C. albicans*. (A)** Schematic representation of the competition between the seryl- (SerRS, violet spheres, PDB accession code 3QNE, Rocha et al., 2011) and leucyl- (LeuRS, red spheres; homology model as described in Table 1) tRNA synthetases (CUG-residues shown as green spheres) for the tRNA(CAG). *C. albicans* tRNA(CAG) model is represented as a white ribbon, and the identity elements for SerRS and LeuRS (acquired during tRNA evolution) are highlighted in blue and red, respectively. The leucine identity elements are mostly localized in the anticodon loop. **(B)** CUG ambiguity in *C. albicans* depends on physiological conditions since tRNA(CAG) is the unique tRNA able to decode the CUG codons. Under normal conditions leucine misincorporation is of approximately 3% and SerRS is the main operating tRNA-synthetase. Under stress conditions, or by genetic manipulation, the fraction of tRNA(CAG) charged with leucine increases, leading to an increase in the percentage of incorporation of leucine at CUG-encoded residues in proteins and resulting in enhanced proteome diversity with consequences in *C. albicans* morphology.

Therefore, in several organisms codon duality is related to the presence of signature motifs in the mRNA that define whether the amino acid is inserted or a terminator tRNA binds to the mRNA, resulting in reassigned codons encoding different amino acids or in truncated proteins, respectively. However, in a particular group of organisms codon duality refers to the ambiguous translation of a codon with arbitrary incorporation of two amino acids depending solely on the relative percentage of tRNAs charged with each of the amino acids. This unique sense-to-sense codon reassignment has been described in the CTG-clade species, which includes the human pathogen *C. albicans* (Tuite and Santos, 1996; Santos et al., 1997; Suzuki et al., 1997). In those species, the universal leucine CUG codon is decoded predominantly as serine, but also as leucine (Ohama et al., 1993; Santos et al., 2011). CUG decoding ambiguity evolved as the result of a mutation in a serine tRNA: the insertion of an adenosine in the anticodon loop of this molecular adaptor generated a mutant serine tRNA with a CAG anticodon (tRNA(CAG)) that matches the CUG codon sequence (Santos et al., 1993). Further changes in the tRNA(CAG) sequence allowed its specific recognition both by seryl- (SerRS) and leucyl-tRNA(LeuRS) synthetases, which aminoacylate this unique tRNA(CAG) with serine or leucine, respectively (Figure 1A). This CUG codon dependent mechanism generates ambiguous proteins and expands the proteome of CTG-clade species (6438 CTG-containing genes can potentially encode 283 billion proteins) (Rocha et al., 2011; Santos et al., 2011), further contributing to the enormous biological complexity of these yeasts, many of which are infectious agents.

## The dual meaning of the CUG codon: a mechanism driving structural and functional proteome plasticity with potential implications in pathogenesis

CUG codons are present in more than half of *C. albicans* coding genes, most of which (~81%) are expressed at low levels (Santos et al., 2011). The majority of the CUG-encoded residues (from hereon named CUG-residues) are present in proteins with functions in plasma and nuclear membranes, Golgi to endosome and Golgi to vacuole transport, DNA repair, regulation of redox homeostasis, DNA replication, chromatin silencing, mRNA splicing, Golgi to plasma membrane transport, mitosis, cytokinesis and protein targeting (Santos et al., 2011).

Comparative proteome analysis of CTG—containing genes in CTG-clade species and related yeasts, showed that ambiguous CUG decoding led to a genome-wide alteration of CUG codon positions: CUG-residues in functionally and structurally relevant positions were mutated to alternative leucine codons (Butler et al., 2009; Rocha et al., 2011). The pressure to avoid excessive accumulation of unfolded proteins, led to the disappearance of the ancestral CUG codons from the genome of CTG-clade species and the introduction of new CUG codons (26 148 codons) with a frequency of 1–38 CTGs per gene, predominantly in positions of low sequence conservation or where serine (about 30% of CUG codons) or other polar amino acid codons were preferred in the open reading frames (ORFs) of related gene products (Butler et al., 2009; Rocha et al., 2011; Santos et al., 2011). A comparative analysis of CTG-containing ORFs suggests that ambiguous decoding is generally well-tolerated since most CUG-residues are partially exposed to the solvent and well distributed between α-helical regions, where leucine is generally favored, and loops, where serine residues are frequently found (Rocha et al., 2011). Evolutionary relocation of CUG-residues to non-conserved regions of the proteins occurred in ~90% of the analyzed gene products. This relocation was apparently driven by the requirement to minimize the impact of codon duality in the structure and function of the affected proteins. In particular, the position of the CUG-residues seems to be optimally suited to prevent the massive protein misfolding that would result from incorporation of polar serine residues in the proteins' hydrophobic cores, where generally leucine residues are favored.

Interestingly, several pieces of evidence favor the hypothesis that, although without drastic structural consequences, the insertion of a serine or leucine within a given position is unlikely to have a completely neutral effect in the overall protein function with consequences in *C. albicans* homeostasis. This is underscored by (i) the frequently observed inability to functionally complement *S. cerevisiae* deletion strains with *C. albicans* CTG-codon containing genes (Feketova et al., 2010), and (ii) the fact that genetic manipulation of *C. albicans* to force an increase in leucine incorporation induces morphological phenotypes, which are associated with the expression of genes involved in cell adhesion and hyphal growth (Gomes et al., 2007).

A striking example of the functional impact of CUG-residue identity is *C. albicans* eukaryotic translation initiation factor 4E (eIF4E), containing a non-conserved CUG-residue (position 166) on the protein surface, that when heterologously expressed in *S. cerevisiae* (where the CUG codon is only translated as leucine) affects the translation rate and the cellular viability of the resulting strain at higher temperatures, in comparison to the wild type yeast (Feketova et al., 2010). Recently, it was also demonstrated that CUG-residue identity has functional consequences in SerRS and LeuRS, the two central players in *C. albicans* CUG codon translation machinery (Rocha et al., 2011; Zhou et al., 2013). These aminoacyl-tRNA synthetases have CUG-residues (Figure 1A) in well conserved regions and insertion of a serine or leucine influences their tRNA aminoacylation activities, with the leucine variants displaying increased enzymatic activity *in vitro* (Rocha et al., 2011; Zhou et al., 2013). Nonetheless, the global effect of these differences in *C. albicans* biology and virulence remains unknown. Although the comparative values for affinities of *C. albicans* SerRS and LeuRS isofoms toward the tRNA(CAG) are still undetermined, under physiological conditions SerRS is the main tRNA(CAG)-charging enzyme and LeuRS appears to be a poor competitor since most of the CUG-residues are translated as serine (Gomes et al., 2007; Santos et al., 2011).

Increasing leucine incorporation levels to 28% by genetic manipulation of *C. albicans*, did not disturb the growth rates but modified cell morphology (Figure 1B) and induced expression of genes involved in cell adhesion and hyphal growth (Gomes et al., 2007), concomitantly with a decreased susceptibility to phagocytosis by murine macrophages (Miranda et al., 2013). Importantly, the incorporation of leucine at CUG positions at levels above 50% leads to dramatic alterations in the expression of genes involved in various metabolic networks that consequently affect *C. albicans* growth rate, phenotype, drug resistance and host immune cell responses (Bezerra et al., 2013). Overall, the identity of the CUG-residue can have functional consequences, even when located on less conserved positions or at the protein surface, influencing post-translational modifications (e.g., phosphorylation) and globally interfering with macromolecular recognition. Therefore, ambiguous insertion of serine or leucine might disturb numerous intracellular processes and contribute to subtle rearrangements of *C. albicans* surface antigens, affecting a vast array of cellular functions with yet unknown consequences. These ambiguous proteins may further constitute a general strategy of the pathogen to increase diversity and survival in varied host niches, mislead/evade the host immune system and accelerate the evolution of drug resistance. Taken together, these data suggest that the levels of serine/leucine incorporation at CUG positions will have widespread consequences in *C. albicans* physiology with potential implications in morphogenesis and host macrophage interactions. Still, the key molecular players linking CUG-residue identity with the observed morphogenetic changes remain so far unidentified.

**Figure 2:**
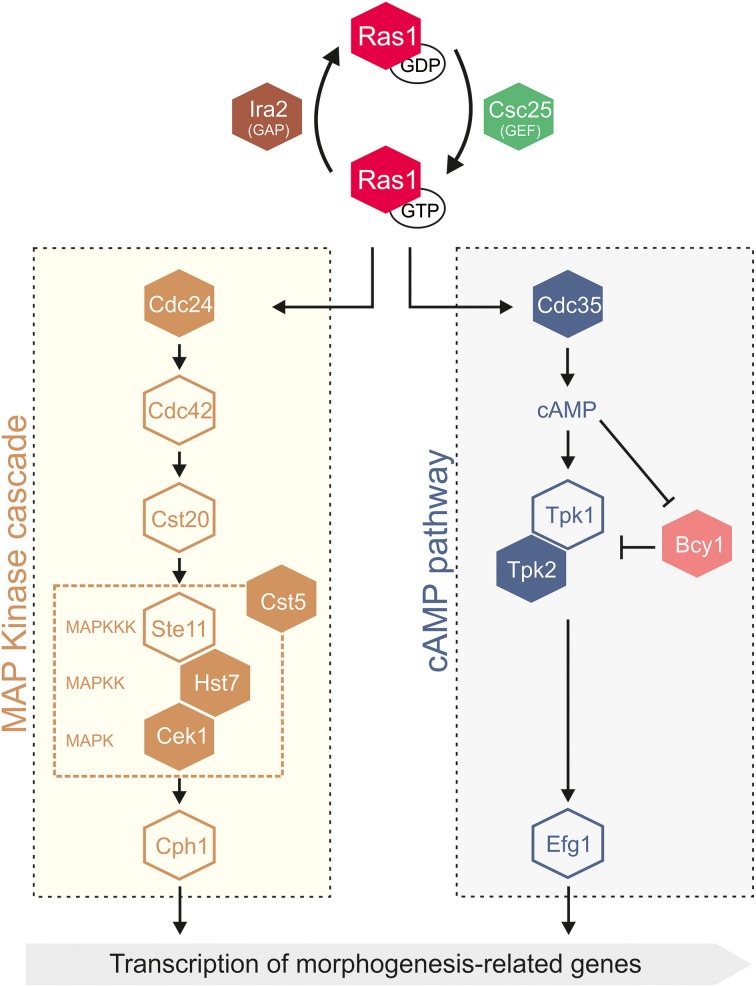
**Scheme of the signal transduction pathways that control *C. albicans* cell cycle and morphogenesis**. Proteins are color coded as follows: GTPases (red), guanidine exchange factor (GEF) (green), GTPase activating protein (GAP) (brown), mitogen-activated protein kinase (MAPK) cascade (light brown), cAMP pathway (blue and pink). MAPK, MAP kinase; MAPKK, MAPK kinase; MAPKKK, MAPKK kinase. Filled boxes correspond to proteins harboring CUG-residues, and therefore subjected to ambiguous decoding.

Curiously, in a small subset of *C. albicans* proteins (10% of CUG-residue containing proteins) CUG-residues are located at conserved active sites, macromolecular recognition sites or in their vicinity, where serine or leucine incorporation could differentially modulate protein function, even without significant structural alterations. In fact, CUG-residues are overrepresented in proteins involved in signal transduction cascades that regulate *C. albicans* cell division, morphogenesis and cell wall composition (Figure 2). Interestingly, some of these proteins are key regulators in cell signaling cascades associated with morphological changes (Csank et al., 1998; Guhad et al., 1998; Feng et al., 1999; Leberer et al., 2001; Bensen et al., 2005; Galán-Díez et al., 2010), which have been widely correlated with *C. albicans* virulence (Alonso-Monge et al., 1999; San-Blas et al., 2000; Romani et al., 2003; Kumamoto and Vinces, 2005; Roman et al., 2007). One possible hypothesis is that a number of CUG-residues might have been strategically selected during evolution to act as molecular sensors, controlling protein function through environmentally-driven fluctuations in serine and leucine incorporation levels (Rocha et al., 2011).

In the following sections the effect of CUG ambiguity will be highlighted in the structure and function of key regulatory proteins associated with the central Ras-dependent signaling pathways (Ras1 GTPase and CEK1 MAP kinase) and the regulation of cell cycle (Cbl2).

## Impact of CUG ambiguity in cell signaling pathways

CUG-residues in conserved positions are overrepresented in components of multi-enzymatic signal transduction cascades, stringently regulated by dynamic protein-protein interactions (Good et al., 2009), such as the Ras1-dependent mitogen-activated protein kinase (MAPK) and cyclic AMP (cAMP)-dependent pathways (Figure 2, CUG-encoded proteins are represented by filled boxes). Two major transcription factors, Cph1 (targeted by MAPK) and Efg1 (targeted by the cAMP-dependent cascade) control the expression of genes required for hyphal growth (Figure 2) (Sudbery, 2011). The signaling cascades respond to environmental stimuli, activating the Ras1 GTPase (one CUG-residue), a branching point in the MAPK and cAMP-dependent pathways (Hogan and Sundstrom, 2009; Sudbery, 2011). Although the molecular details of Ras1 activation remain elusive, its direct association to the adenylyl cyclase Cyr1 (or Cdc35, four CUG-residues), the only adenylyl cyclase described in *C. albicans*, is essential for cAMP synthesis. The cAMP-dependent PKA complex with the catalytic subunits Tpk1 and Tpk2 (four CUG-residues) and the regulatory subunit Bcy1 (one CUG-residue) (Giacometti et al., 2012), activates transcription factors regulating the expression of virulence genes (e.g., *ALS3*, *HWP1*) (Shapiro et al., 2011). The filamentous growth MAPK cascade responds to nutrient restriction targeting the transcription factor Cph1. It includes the kinase Cst20, the MAPKKK Ste11, the MAPKK Hst7 (five CUG-residues), the scaffold protein Cst5 (three CUG-residues) and the MAPK Cek1 (one CUG-residue) (Figure 2). This MAPK pathway was recently shown to be reprogrammed within the CTG-clade, where adjustments occurred in the scaffold protein Cst5, that is significantly smaller than its *S. cerevisiae* ortholog (Cõte et al., 2011).

### Ras1—a central GTPase at the crossroad between MAPK and c-AMP signaling pathways

Ras is a highly conserved GTPase that acts as a molecular sensor of environmental stress signals, such as temperature and nutrient deprivation, regulating two downstream signaling cascades, the MAPK pathway and the cAMP-dependent pathway (Figure 2). Ras is a highly dynamic GTP hydrolysing enzyme, whose conformation switches between active GTP-bound and inactive GDP-bound state (Bourne et al., 1991). Two conserved motifs in the proximity of the catalytic site are critical for GTP hydrolysis, Switch I and Switch II (Campbell et al., 1998). While Switch I is crucial for GDP release and suffers significant conformational changes required for the release of the bound nucleotide, Switch II motif is responsible for the activation of the catalytic water required for GTP hydrolysis (Pai et al., 1990; Tong et al., 1991; Buhrman et al., 2010).

**Figure 3:**
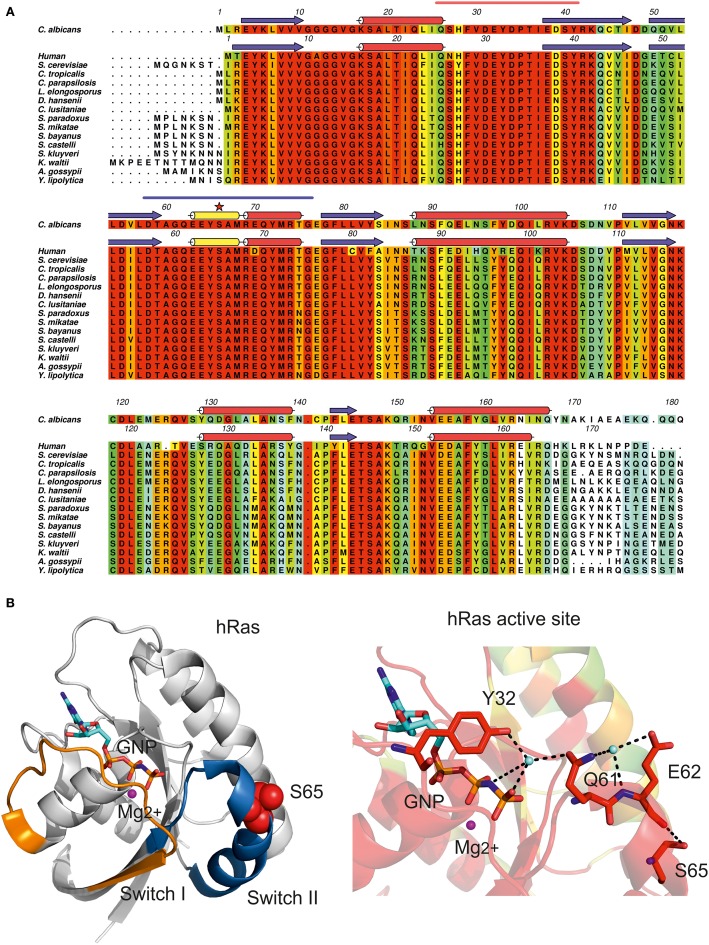
**Impact of CUG ambiguity in *C. albicans* Ras1 GTPase. (A)** Amino acid sequence alignment of *C. albicans* Ras1 orthologs and human Ras. Residues are colored according to conservation scale (red: identical residues, orange to blue: decreasing scale of conservation of amino acid properties in each alignment column; white: dissimilar residues). *C. albicans* Ras1 secondary structure elements based on Ras1 homology model (see Table 1) are represented above the corresponding amino acid sequence. Human Ras secondary structure elements based on human Ras three-dimensional structure (Buhrman et al., 2010; PDB accession code 3K8Y) are represented above the corresponding amino acid sequence (red cylinders, α-helices; yellow cylinders, 3_10_ helices; blue arrows, β-sheets); the CUG-residue is marked by a red star and Switch I and Switch II motifs are marked by orange and blue lines, respectively. Figure prepared with Aline (Bond and Schuttelkopf, 2009). **(B)** Cartoon representation of human Ras (hRas, white). Switch I and Switch II regions are colored in orange and blue, respectively. hRas residue Ser65 is represented as red spheres. **(C)** Detailed view of hRas catalytic site. Ser65 is structurally equivalent to *C. albicans* Ras1 CUG-residue 66. Residues are colored according to residue conservation as shown in **(A)**. Selected residues are shown as sticks. Hydrogen bonds are represented as dashed black lines. The magnesium ion is represented as a magenta sphere and cyan spheres represent ordered water molecules. Nonhydrolyzable GTP analog (GNP) is represented in cyan (carbon atoms). **(B,C)** Prepared with PyMOL (http://www.pymol.org).

*C. albicans* Ras1 shares 67% amino acid sequence identity with the well-characterized human Ras (hRas), for which the three-dimensional structure of the catalytic domain is known (Buhrman et al., 2010) (Table 1). In *C. albicans*, the CUG-residue at position 66 is strictly conserved and structurally equivalent to hRas Ser65 (Figure 3A, red star). This residue is positioned at the surface of the mobile Switch II region (Figure 3B, red spheres) being involved in a network of polar interactions that bridge Switch II and Switch I, which is strictly conserved in *C. albicans* Ras1 (Figure 3C). In particular, Ser65 hydrogen bonds to the main-chain carbonyl of Glu62, contributing to maintain the optimal conformation of Switch II loop residues (including the catalytic Gln61), which are required for stabilization of the nucleotide γ-phosphate (Figure 3C). Therefore, the exchange of serine for leucine at the CUG position of *C. albicans* Ras1 is predicted to partly destabilize this hydrogen bond network, which might result in alterations in the conformation/mobility of the Switch II loop and impact Ras1 catalytic activity. In fact, further support for the relevance of this polar interaction network for Ras activity stems from analysis of human (hRas) or *S. cerevisiae* Ras1 homologs, where it was shown that Gln61 mutations lock the protein in the active GTP-bound conformation, resulting in transiently activated phenotypes (Buhrman et al., 2007).

**Table 1:** **Overview of analyzed *C. albicans* proteins**.

**Biological processes**	**Protein**	**CUG-residue**	**Template structure (PDB accession code)^#^**	**Amino acid sequence identity (%)**	QMEAN score^*^
Protein translation	LeuRS	919	*T. termophilus* isoleucyl-tRNA synthetatse (1JZQ)	34	0.29
Ras signaling	Ras1	66	Human Ras (3K8Y)	67	0.76
	Csc25 (GEF)	939; 1059	Human Sos1-Ras complex (1BKD)	32	0.60
	Ira2 (GAP)	1312; 1383; 1397	Human Ras-GAP334 complex (1WQ1)	30	0.53
MAPK pathway	Cek1	199	*S. cerevisiae* Fus3 (2B9F)	64	0.70
Cell cycle	Clb2	301; 383	Human cyclin B1 (2B9R)	39	0.66
	Cdc28	–	Human Cdk2-Cyclin B1 complex (2JGZ)	67	0.77

# *The selected experimental three-dimensional structures were used as templates in SWISS-MODEL (http://swissmodel.expasy.org/) (Arnold et al., 2006) for calculating homology models of C. albicans proteins, from which secondary structure elements were inferred and used in sequence alignments shown in Figures 3A, **5A**, **6A,D***.

* *The quality of homology models was analyzed with the Qualitative Model Energy ANalysis (QMEAN) server (Benkert et al., 2008, 2009). The QMEAN score is a composite score consisting of a linear combination of six terms: Cβ interaction energy, all-atom pairwise energy, solvation energy, torsion angle energy, secondary structure agreement (SSE_agree) and solvent accessibility agreement (ACC_agree). The value associated to the QMEAN-score ranges between 0 and 1 with higher values for more reliable models. C. albicans LeuRS model is of insufficient quality for detailed structural analysis and was exclusively used for the schematic representation shown in Figure 1A*.

**Figure 4:**
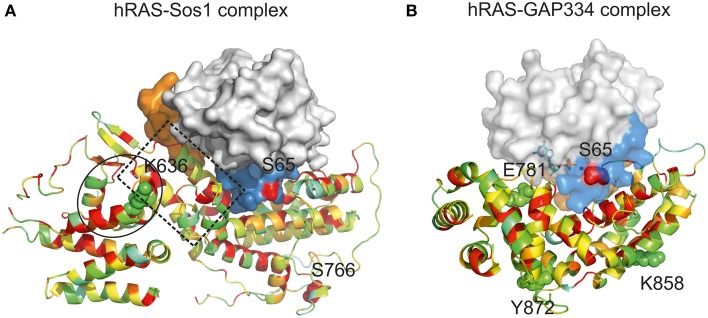
**Impact of CUG ambiguity in recognition of *C. albicans* Ras1 by GAPs and GEFs. (A)** Representation of hRas-Sos1 complex (Boriack-Sjodin et al., 1998; PDB accession code 1BKD). hRas is represented as a solid surface in which Switch I region is colored in orange, Switch II region is colored in blue, and residue Ser65 is represented as red spheres. Sos1 is represented as a cartoon colored according to residue conservation with *C. albicans* Csc25. Residues K636 and S766 (structurally equivalent to *C. albicans* CUG-residues Ser939 and Ser1059) are represented as spheres. The helical hairpin of the Sos1 catalytic domain is highlighted by a dashed box. The Sos1 N-terminal region responsible for the stabilization of the helical hairpin is within a black circle. **(B)** Representation of hRas-GAP334 complex (Scheffzek et al., 1997; PDB accession code 1WQ1). hRas representation as in **(A)**. GAP334 is represented as a cartoon colored according to residue conservation with *C. albicans* Ira2. Residues Glu781, Lys858, and Tyr872 (structurally equivalent to *C. albicans* CUG-residues 1312, 1383, and 1397) are represented as spheres. Figures were prepared with PyMOL (http://www.pymol.org).

Ras GTPase activity is intrinsically low and physiologically regulated by interaction with two distinct families of proteins: positive regulators (Guanidine Exchange Factors, GEFs), and negative regulators (GTPase Activating Proteins, GAPs) (Boguski and Mccormick, 1993). GEF proteins enhance Ras activity by promoting the release of bound GDP and the uptake of new GTP (Boriack-Sjodin et al., 1998) in response to the upstream stimuli. In contrast, GAP proteins accelerate the slow intrinsic rate of GTP hydrolysis by several orders of magnitude (Scheffzek et al., 1997). Importantly, the Ras Switch regions are central for recognition by GEFs and GAPs (Figures 4A,B), further suggesting a role for CUG-residue identity in *C. albicans* Ras1-GTP recycling.

*C. albicans* Csc25 displays 32% amino acid sequence identity with the guanine-nucleotide-exchange-factor region of the human GEF, Son-of-sevenless 1 (comprising residues 564–1049, referred to as Sos1), for which the three-dimensional structure in complex with hRas is known (Boriack-Sjodin et al., 1998) (Table 1). The structure of the Sos1-hRas complex revealed that Sos1 is composed of two distinct α-helical domains. While the Sos1 C-terminal domain comprises the hRas interaction region, the N-terminal region displays predominantly a structural role, contributing for the stabilization of the helical hairpin that inserts into hRas active site, forcing the displacement of Switch II region, occupying the magnesium binding site and triggering nucleotide exchange (Figure 4A). The functionally important features of Sos1 structure appear to be preserved in Csc25, and the interaction surface is relatively well conserved. Particularly relevant is the conservation of the residues at the interface between Csc25 and Ras1, where Switch II plays a central role (Boriack-Sjodin et al., 1998) (Figure 4A). In the Sos1-hRas complex, most surface residues of Switch II interact with hRas (Boriack-Sjodin et al., 1998). Considering the tight interaction between Sos1 and hRas, it is predictable that the modification of the conserved serine by a leucine at position 66 in *C. albicans* Ras1 (topologically equivalent to position 65 in hRas) will interfere with Csc25 recognition with consequences for GDP/GTP exchange. Interestingly, Csc25 contains four CUG-residues, two of them located within the conserved nucleotide-exchange-factor region (positions 939 and 1059, structurally equivalent to Sos1 Lys636 and Ser766) (Figure 4A, spheres). In particular, the CUG-residue at position 939 in *C. albicans* Csc25 is located in the structural N-terminal region, adjacent to the topologically equivalent helical hairpin in Sos1. Therefore, it is predictable that modification of CUG-residue identity at this position within Csc25 might additionally affect its interaction with Ras1, further modulating the complex mechanisms of molecular recognition in this Ras-dependent signaling pathway.

*C. albicans* Ira2 is the GAP responsible for repression of Ras1 activity (Nobile et al., 2012). Ira2 displays 30% amino acid sequence identity with the catalytic region of human GAP, p120^GAP^ (residues 714–1047, from hereon named GAP334), for which the three-dimensional structure in complex with hRas has been determined (Scheffzek et al., 1997) (Table 1). hRas binds to the shallow groove of GAP334, and interaction results in the stabilization of hRas Switch II (Scheffzek et al., 1997). The interaction interface between GAP334 and hRas is well conserved in Candida species orthologs, particularly in the proximity of Ras1 Switch II region (Figure 4B). In this region, Switch II is stabilized by a combination of polar and hydrophobic interactions involving invariant residues such as Tyr64, Glu62 and Gln61 from hRas (equivalent to Tyr65, Glu63 and Gln62 in *C. albicans* Ras1), and Leu902, Arg749, and Arg789 in GAP334 (equivalent to Leu1424, Asn1281, and Arg1320 in Ira2). Importantly, the conserved interaction between hRas Gln61 and GAP334 Arg789, poises the side chain of the catalytic residue for optimal GTP hydrolysis. Considering that the Ras1 CUG-residue is located in the proximity of the Ras1-Ira2 interface, the presence of a leucine or a serine at this region is likely to modify the tight packing between these two proteins and interfere with the GAP-modulated activation of Ras1 GTPase activity. Curiously, Ira2 contains 11 CUG-residues, from which only three (1312, 1383, and 1397) are located within the catalytic and structurally characterized region of GAP and are structurally equivalent to GAP334 Glu781, Lys858, and Tyr872 (Figure 4B, spheres). All these CUG-residues are predicted to be located at the protein surface and primarily in non-conserved positions, although the full impact of CUG identity for the assembly and function of the GAP-Ras complex remains to be established.

In *C. albicans*, Ras1-dependent pathways regulate the downstream activity of transcription factors Cph1 and Efg1 that control the expression of genes responsible for the tight control of morphogenesis and virulence (Figure 2) (Leberer et al., 1997, 2001; Feng et al., 1999). In fact, in response to nitrogen starvation *C. albicans* Ras1 is activated through a transmembrane ammonium permease receptor (Mep) controlling yeast-to-hypha morphogenesis through the activation of *C. albicans* polarized growth (Shapiro et al., 2011). Deletion of *C. albicans RAS1* gene leads to an early entry in stationary phase, increased resistance to H_2_O_2_, higher sensitivity to heavy metal Co^2+^ (Zhu et al., 2009), repression of CO_2_-induced white-to-opaque switching (Huang et al., 2009) and to the inhibition of serum-induced germ tubes and hyphae formation (Feng et al., 1999). The link between Ras1 and morphogenesis is highlighted by the identification of point mutations in *RAS1* gene directly affecting *C. albicans* morphology. In particular the Gly-to-Val mutation at position 13, within the Ras1 active site, leads to a dominant active Ras1 with consequent increased hyphal growth. On the other hand, the mutation of Gly16 to Ala16, also at the Ras1 active site, represses Ras1 activity and *C. albicans* hyphal growth (Feng et al., 1999). So far, experimental evidence providing a direct link between CUG ambiguity in the molecular machinery regulating Ras1 GTPase activity and their influence on *C. albicans* virulence is still lacking. However, this structural analysis shows that a conserved CUG-residue is located in the functional Switch II region of Ras1, close to the GAP and GEF interaction surface, hinting that Ras1 CUG-residue identity might affect the flow of the downstream MAPK- and cAMP-dependent protein kinase pathways, ultimately explaining the morphological changes observed in engineered *C. albicans* cells upon increased leucine incorporation (Gomes et al., 2007).

### Cek1 MAP kinase

**Figure 5:**
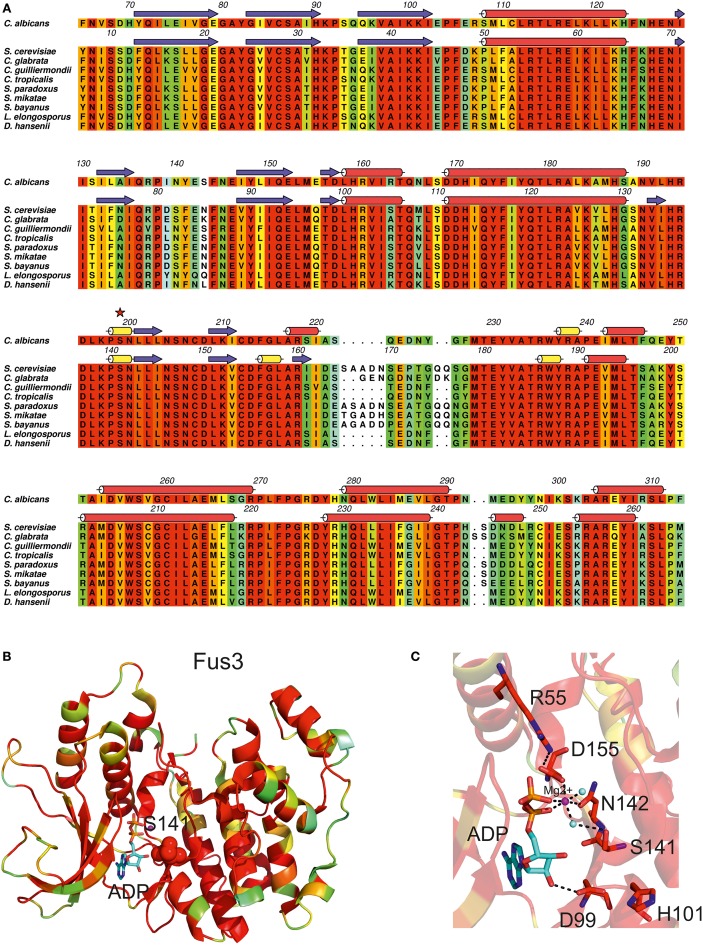
**Impact of CUG ambiguity in *C. albicans* Cek1 kinase. (A)** Amino acid sequence alignment of *C. albicans* Cek1 orthologs. Residues are colored according to a residue conservation scale (red: identical residues, orange to blue: decreasing conservation of amino acid properties; white: dissimilar residues). *C. albicans* Cek1 secondary structure elements based on a homology model (see Table 1) are represented above the corresponding amino acid sequence and the CUG-residue is marked by a red star. *S. cerevisiae* Fus3 secondary structure elements (Remenyi et al., 2005; PDB accession code 2B9F) are represented above the corresponding amino acid sequence (red cylinders, α-helices; yellow cylinders, 3_10_ helices; blue arrows, β-sheets). Figure prepared with Aline (Bond and Schuttelkopf, 2009). **(B)** Cartoon representation of *S. cerevisiae* Fus3 structure (Remenyi et al., 2005; PDB accession code 2B9F) colored according to residue conservation as shown in **(A)**. Fus3 residue Ser141 (structurally equivalent to *C. albicans* Cek1 CUG-residue 199) is represented as red spheres. **(C)** Detailed view of the ATP-binding pocket of *S. cerevisiae* Fus3. Ser141 stabilizes a water molecule that coordinates the magnesium ion (Mg^2+^), contributing to the stabilization of the γ-phosphate of the bound nucleotide. Cartoon and stick representation of Fus3 colored according to conservation as shown in **(A)**. Selected residues are shown as sticks. Hydrogen bonds are represented as dashed lines. The magnesium ion is represented as a magenta sphere and cyan spheres represent ordered water molecules. The active site-bound nucleotide is represented with sticks (carbon atoms colored in cyan). **(B,C)** Prepared with PyMOL (http://www.pymol.org).

The *C. albicans* MAP kinase Cek1, displays an invariant CUG-residue at position 199 (Figure 5A). This MAPK shares 58% of amino acid sequence identity with *S. cerevisiae* Fus3, for which the crystal structure has been determined (Remenyi et al., 2005) (Table 1). The CUG-residue is structurally equivalent to Fus3 Ser141 (Figure 5B, red sphere), located within a conserved polar pocket at the ATP binding cleft, and stabilizing a magnesium-bound water molecule (Figure 5C). Insertion of a leucine at position 199 would be highly unfavorable leading to the destabilization of magnesium coordination and potentially interfering with nucleotide binding. Importantly, the MAPK pathway, highly dependent on protein-protein interactions for signal transduction, is also highly enriched in proteins containing CUG-residues (Figure 2). Curiously, several facts point to an interesting remodeling of the molecular interaction networks associated with the MAPK signal transduction pathways in *C. albicans* (Stynen et al., 2010). Despite the high amino acid sequence identity between *C. albicans* proteins and their orthologs from *S. cerevisiae*, recent data showed that the disappearance of the Fus3-binding vWA (von Willebrand type A) domain from the scaffold protein Cst5 reshaped the protein-protein interactions in this essential signaling pathway (Zalatan et al., 2012). The relevance of CUG-residue identity for reshaping the molecular surface of the proteins associated in this interaction network is highlighted by discrepancies between the Cek1 interaction networks identified by yeast-two-hybrid assays in *S. cerevisiae* (where all CUG codons are translated as leucine) (Cõte et al., 2011) and *C. albicans* (Stynen et al., 2010). Therefore CUG-residue decoding as a serine might be required for correct docking interactions to occur, and insertion of leucine at these positions is expected to interfere with the delicate balance between protein interactions that leads to target protein phosphorylation and the correct signal transduction flow.

In *C. albicans* distinct stimuli such as starvation, nitrogen deprivation, oxidative and osmotic stress, exposure to serum or H_2_O_2_, increased temperature and low pH activate cell signaling cascades such as the MAPK pathway (Figure 2). The MAPK cascade targets the downstream transcription factor Cph1, which controls the expression of genes required for morphological switching (Sudbery, 2011; Yi et al., 2011) cell wall integrity, host recognition (Galán-Díez et al., 2010), pheromone-activated mating and biofilm formation (Cõte et al., 2011; Yi et al., 2011). A previous report showed that *C. albicans CEK1* homozygous null mutants display lower ability to develop hyphae, which is reverted once *CEK1* gene is reintegrated in the genome, suggesting an important role for Cek1 in the control of morphogenesis (Csank et al., 1998). Moreover, Cek1 appears to confer resistance to cell wall disturbing agents, namely Calcofluor White and Congo Red, and to reduce flocculation (Li et al., 2009). Since host-pathogen interactions are mediated mainly through *C. albicans* cell wall, whose composition is regulated by the Cek1-mediated MAPK pathway (Csank et al., 1998; Sudbery, 2011), this kinase plays a crucial role in *C. albicans* pathogenicity.

Although the full consequences of these MAPK-activating stimuli on the relative Ser/Leu insertion at CUG-encoded sites is still undisclosed, the structural analysis of the Cek1 CUG-residue position reveals that an increased insertion of leucine at this location is expected to have a functional impact. Studying the molecular interactions regulating this MAPK and how they are shaped by CUG-residue identity will likely provide relevant data to further understand the impact of this codon reassignment for *C. albicans* cell wall composition, morphogenesis and virulence.

### Ambiguous CUG decoding in Clb2, an essential cyclin in *C. albicans* cell cycle

**Figure 6:**
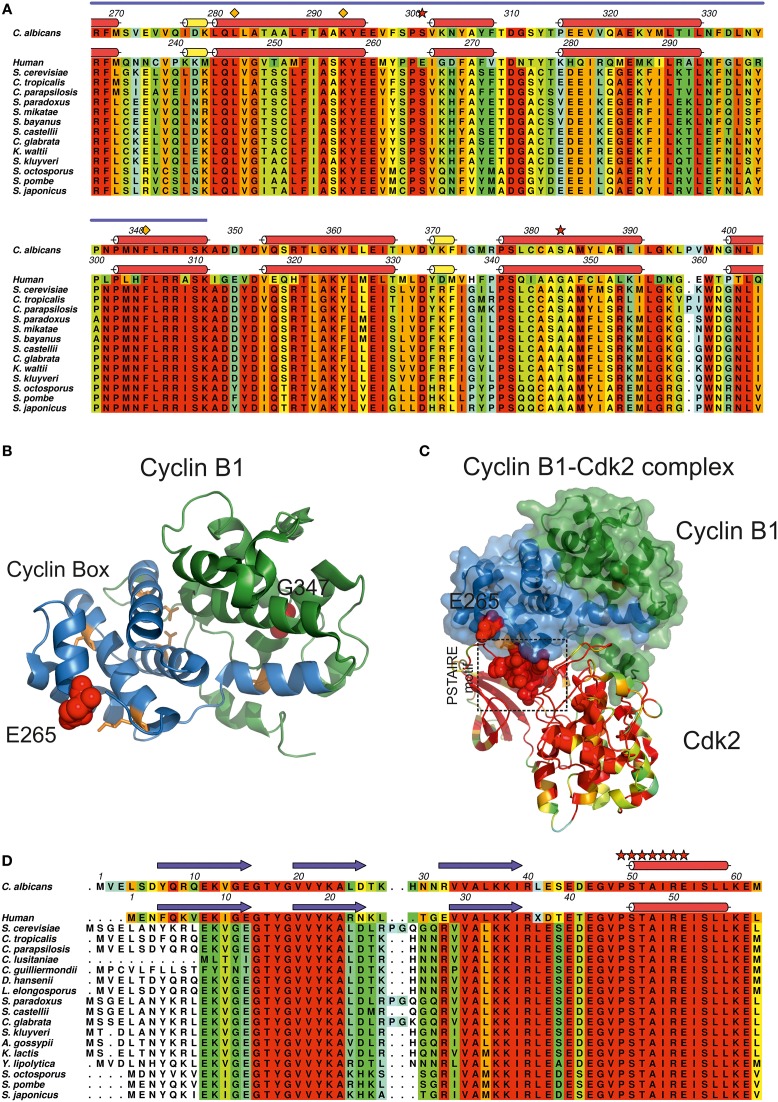
**Impact of CUG ambiguity in *C. albicans* cyclin Clb2. (A)** Amino acid sequence alignment of *C. albicans* Clb2 orthologs and human cyclin B1. Residues are colored according to a residue conservation scale (red: identical residues, orange to blue: decreasing conservation of amino acid properties; white: dissimilar residues). Relevant residues are depicted above the alignment (orange diamonds: two of the well-spaced residues present in the so-called cyclin-box (RDLKF) conserved from yeast to humans; red stars: *C. albicans* Clb2 CUG-residues 301 and 383. Secondary structure elements of a *C. albicans* Clb2 homology model (see Table 1) are represented above the corresponding amino acid sequence. Human cyclin B1 secondary structure elements (Petri et al., 2007; PDB accession code 2B9R) are represented above the corresponding amino acid sequence (red cylinders, α-helices; yellow cylinders, 3_10_ helices). Cyclin box region is indicated by a blue line above the alignment. **(B)** Cartoon representation of human cyclin B1 structure (Petri et al., 2007; PDB accession code 2B9R). Cyclin B1 cyclin box region is colored in blue, conserved residues within the cyclin box (RDLKF) are colored in orange. Residues Glu265 and Gly347 (structurally equivalent to *C. albicans* Clb2 CUG-residues 301 and 383) are represented as red spheres. **(C)** Representation of the human cyclin B1-Cdk2 complex (Brown et al., 2007, PDB accession code 2JGZ). Human cyclin B1 is represented as a solid surface with an underlying cartoon colored as in **(B)**, highlighting the two central subdomains. Human Cdk2 is represented as a cartoon colored according to residue conservation with *C. albicans* Cdc28. The conserved Cdk2 PSTAIRE motif is represented as red spheres. **(D)** Amino acid sequence alignment of *C. albicans* Cdc28 orthologs and human Cdk2. Residues are colored according to a residue conservation scale (red: identical residues, orange to blue: decreasing conservation of amino acid properties; white: dissimilar residues). Secondary structure elements of a *C. albicans* Cdc28 homology model (see Table 1) are represented above the corresponding amino acid sequence. Human Cdk2 secondary structure elements (Brown et al., 2007; PDB accession code 2JGZ) are represented above the corresponding amino acid sequence (red cylinders, α-helices; yellow cylinders, 3_10_ helices; blue arrows, β-sheets). Relevant residues are depicted above the alignment (red stars: PSTAIRE motif of Cdk2 which is conserved from yeast to humans). **(A,D)** Prepared with Aline (Bond and Schuttelkopf, 2009). **(B,C)** Prepared with PyMOL (http://www.pymol.org).

*C. albicans* cyclin Clb2, an essential B-type cyclin harboring two CUG-residues, one of which at a strictly conserved site (Figure 6A, red stars), is involved in the regulation of cell cycle progression and morphogenesis (Lew and Reed, 1993; Bensen et al., 2005) through binding/activation of cyclin-dependent kinases (CDKs). In *C. albicans*, the expression and function of cyclins appears to be morphology-dependent: while some cyclins perform their function merely in the yeast form, others are only active throughout the hyphal form (Lew and Reed, 1993; Loeb et al., 1999; Bensen et al., 2005; Sinha et al., 2007; Cõte et al., 2011). In fact, Clb2 levels are cell cycle-dependent, peaking during G2/M phase and decreasing before the mitotic exit. Clb2 is more abundant in the yeast form and the accumulation of Clb2 is delayed upon hyphal induction, suggesting that its expression is tightly regulated during morphogenesis (Bensen et al., 2005). While the disruption of *CLB2* leads to cell cycle arrest in late anaphase, increasing cell elongation and the appearance of divided nuclei connected by long mitotic spindles, the overexpression of *CLB2* reverts this phenotype reducing the extent of filamentous growth (Bensen et al., 2005). The CUG-residue 301 present at a Clb2 conserved site, is within the so-called cyclin box domain (Figures 6A,B), an interaction interface of about 100 residues containing five well-spaced conserved residues (RDLKF) (Nugent et al., 1991; Kobayashi et al., 1992) (Figure 6A, orange diamonds) and a structural motif of five α-helices, the “cyclin fold,” highly conserved from yeast to humans. *C. albicans* Cbl2 shares 39% amino acid sequence identity with human cyclin B1 (Table 1, Figure 6A), for which the experimental three-dimensional structure is known (Petri et al., 2007). *C. albicans* Clb2 CUG-residues (301 and 383) are structurally equivalent to amino acids Glu265 and Gly347 in human cyclin B1 (Figure 6A red stars, Figure 6B red spheres). In particular, cyclin B1 Glu265 (invariant serine in all yeast homologs, Figure 6A) is exposed and in a loop between two α-helices within the conserved cyclin box (Figure 6B, blue cartoon) where the interaction with the activating kinase, CDK, occurs.

CDKs are proline-directed kinases that phosphorylate serines or threonines for the control of cell cycle progression. In most eukaryotes, activation of CDKs is mediated by transcriptional and post-transcriptional mechanisms (Duronio and Xiong, 2013), such as the phosphorylation of a threonine residue, present at a T-loop of CDKs structurally close to the PSTAIRE motif interaction region (Figures 6C,D red stars) that opens the protein substrate binding region and favors the contact/interaction between the PSTAIRE motif of CDKs and the cyclin box region from cyclins (Hanks and Hunter, 1995; Kaldis, 1999; Ross et al., 2000; Brown et al., 2007) (Figure 6C). In *C. albicans*, the best studied CDK is Cdc28 due to its central role in the coordination of yeast cell cytokinesis, cycle progression (Murray, 2004) and cycle-related morphogenesis (Umeyama et al., 2006). Repression of *CDC28* affects the expression of transcription factors that regulate morphogenesis, namely through the control of the expression levels of *EFG1*, *NRG1*, *RBF1*, *RIM101*, *FKH2*, and *TEC1*, leading to highly polarized filamentous growth of *C. albicans* cells (Umeyama et al., 2006). Cdc28 displays high sequence similarity to human Cdk2 (66% identity) (Table 1, Figure 6D), particularly within the cyclin interacting PSTAIRE region (Figure 6D, red stars). Therefore, the presence of a larger aliphatic leucine at position 301 in Clb2 may affect the interaction of Clb2 with Cdc28 suggesting a role for ambiguity in the regulation of protein–protein interactions required for cyclin binding and CDK activation.

Altogether, these observations strongly suggest a role for CUG ambiguity in the control of the stability/strength of this crucial cyclin-CDK complex, whose association is essential for Cdc28 activation. Consequently, CUG-residue identity may influence the function of the cyclin-CDK complex in the control of cell cycle and morphogenesis, ultimately affecting *C. albicans* cellular homeostasis and pathogenesis.

## Conclusion

Throughout evolution, different species developed divergent strategies to adapt to the constantly changing environmental conditions. In particular, yeast of the CTG-clade species underwent a unique sense-to-sense codon reassignment: the CUG codon is ambiguously translated both as serine and leucine, leading to proteome diversity (Santos et al., 1996; Tuite and Santos, 1996). Interestingly, under physiological conditions most of the CUG codons are translated as serine and specific stimuli lead to increased leucine incorporation (Bezerra et al., 2013). The increase of CUG ambiguity induces phenotypical alterations in CTG-clade species, such as morphological changes, widely correlated with fungal virulence (Roman et al., 2007). However, at the molecular level little is known about the impact of CUG ambiguity in the structure/function of the affected proteins and its relevance for the observed phenotypical changes. Although the genome in these species has evolved to tolerate genetic code ambiguity and moderate fluctuations in the insertion of serine or leucine at CUG positions, some proteins involved in key signal transduction pathways are likely to be functionally affected by the identity of the CUG-residue. The structural analysis here presented reveals that increased insertion of leucine at CUG sites in key proteins such as Ras1, Cek1, and Cbl2, are not expected to affect overall protein structure dramatically. Instead, it is likely that CUG ambiguity interferes with protein function, particularly with the assembly and stability of macromolecular complexes crucial for the regulation of essential cellular processes. The fine modulation of the activity of a number of selected *C. albicans* proteins, mediated by CUG ambiguity, may be an effective evolutionary strategy to adapt to different hostile environments.

The structural analysis of this small sample of key regulatory proteins suggests that the incorporation of a small polar serine or a longer aliphatic leucine at the CUG position might have an effect on the protein interfaces and modulate protein-protein interaction dynamics. Therefore, a role for CUG ambiguity in the regulation of *C. albicans* morphogenesis/pathogenesis is suggested and should be further explored, at the molecular level, as an important virulence regulatory process and a target for new therapeutic strategies.

### Conflict of interest statement

The authors declare that the research was conducted in the absence of any commercial or financial relationships that could be construed as a potential conflict of interest.
